# Adrenal Rest Tumor of the Liver Preoperatively Diagnosed as Hepatocellular Carcinoma

**DOI:** 10.1155/2017/8231943

**Published:** 2017-06-19

**Authors:** Megumu Enjoji, Katsuya Sanada, Ryota Seki, Takashi Ito, Masato Maeda

**Affiliations:** ^1^Department of Surgery, Mishima General Hospital, 2276 Yata, Mishima, Shizuoka 411-0801, Japan; ^2^Department of Human Pathology, Tokyo Medical and Dental University Graduate School, 1-5-45 Yushima, Bunkyo-ku, Tokyo 113-8519, Japan; ^3^Department of Gastrointestinal Medicine, Mishima General Hospital, 2276 Yata, Mishima, Shizuoka 411-0801, Japan

## Abstract

**Background:**

Hepatic adrenal rest tumors are rare and show similar findings to hepatocellular carcinoma (HCC). It is difficult to distinguish an adrenal rest tumor from HCC due to radiological similarity. We report a case of an adrenal rest tumor in the liver that mimicked HCC radiologically.

**Case Presentation:**

A 67-year-old female was referred to our hospital due to the finding of a hepatic mass. Enhanced computed tomography revealed a 17 mm well-defined tumor that was enhanced in the arterial phase and washed out in the portal and delayed phase in the posterosuperior subsegment of the right hepatic lobe, and HCC was suspected. We performed a subsegmental resection of the liver. Microscopic findings showed that the tumor was composed of pale cells, and tumor cells were aligned in alveolar or fascicular arrangements in a similar manner to features of adrenocortical tissue. Immunohistochemically, the tumor expressed synaptophysin and CD56. The final histopathologic diagnosis in this case was an adrenal rest tumor of the liver.

**Conclusions:**

An adrenal rest tumor is similar to HCC in radiological findings. This hepatic tumor should be added to the list of radiological differential diagnoses of hypervascular hepatic tumors.

## 1. Background

Adrenal rest tumors are rare tumors commonly located around the kidney, retroperitoneum, spermatic cord, para testicular region, and broad ligament [1, 2]. Adrenal rest tumors are collections of aberrantly located adrenocortical tissues, and they rarely occur in the liver. We searched in the PubMed database and found only nine case reports of hepatic adrenal rest tumor [3–11]. Two cases were described to be associated with endocrine abnormalities and malignant histological feature [3, 4]. Seven cases were nonfunctional, presenting as incidental findings [5–11]. These seven tumors were discovered in the posterosuperior subsegment of the right hepatic lobe (segment 7), and HCC could not be excluded preoperatively. Adrenal rest tumors in the liver have been reported to appear as a fat containing and hypervascular mass on imaging [6]. Radiologically, adrenal rest tumors show similar findings to hepatocellular carcinoma (HCC) or angiomyolipoma. It is difficult to distinguish an adrenal rest tumor from HCC or angiomyolipoma due to radiological similarity.

Here, we report a case of an adrenal rest tumor in the liver that mimicked HCC radiologically.

## 2. Case Presentation

A 67-year-old female was referred to our hospital due to the finding of a hepatic mass after an ultrasonography (US) for elevated serum gamma-glutamyl transpeptidase (*γ*-GTP). Her past medical history was positive for diabetes for the last seven years, which was well-controlled by oral hypoglycemic drugs. Her family history was noncontributory. The laboratory investigation showed no elevation of serum alpha-fetoprotein (AFP) or protein induced by vitamin K absence/antagonist-II (PIVKA-II). Hepatitis B surface antigens and hepatitis C antibodies were negative. The aspartate aminotransferase (AST) was 21 IU/L, alanine aminotransferase (ALT) was 20 IU/L, alkaline phosphatase (ALP) was 184 IU/L, and *γ*-GTP was 109. The total bilirubin level was 0.75 mg/dL, the albumin level was 4.6 g/dL, and the prothrombin time was 12.0 s (INR, 0.93).

**Figure 1:**
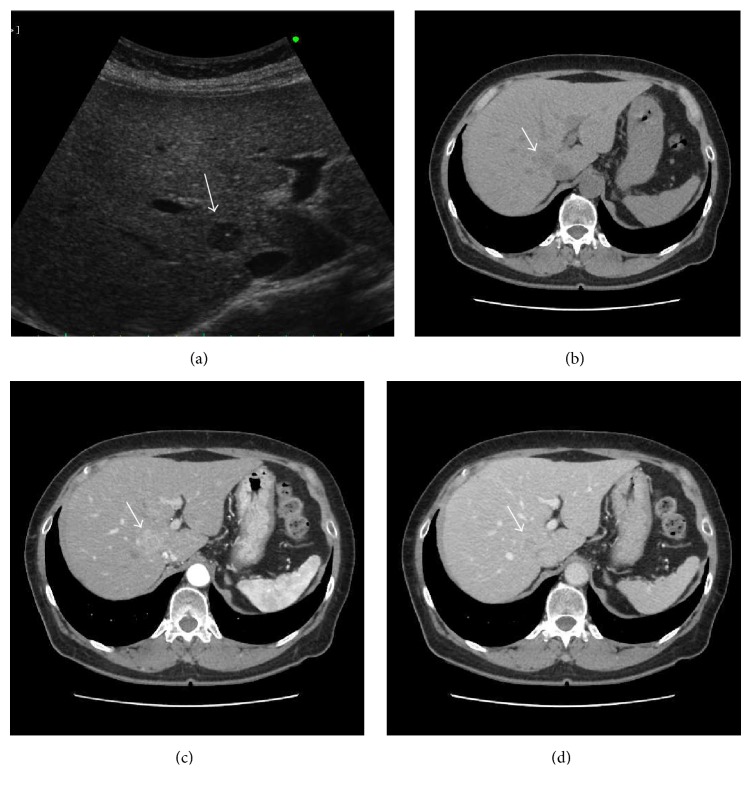
Ultrasonography and computed tomography. US revealed a 14 × 10 mm well-defined, hypoechoic mass (arrow) in the posterosuperior subsegment of the right hepatic lobe (segment 7) (a). Plain CT shows a low-density mass at the right hepatic lobe (arrow) (b). The tumor was enhanced in the arterial phase (c) and washed out in the portal phase (d).

**Figure 2:**
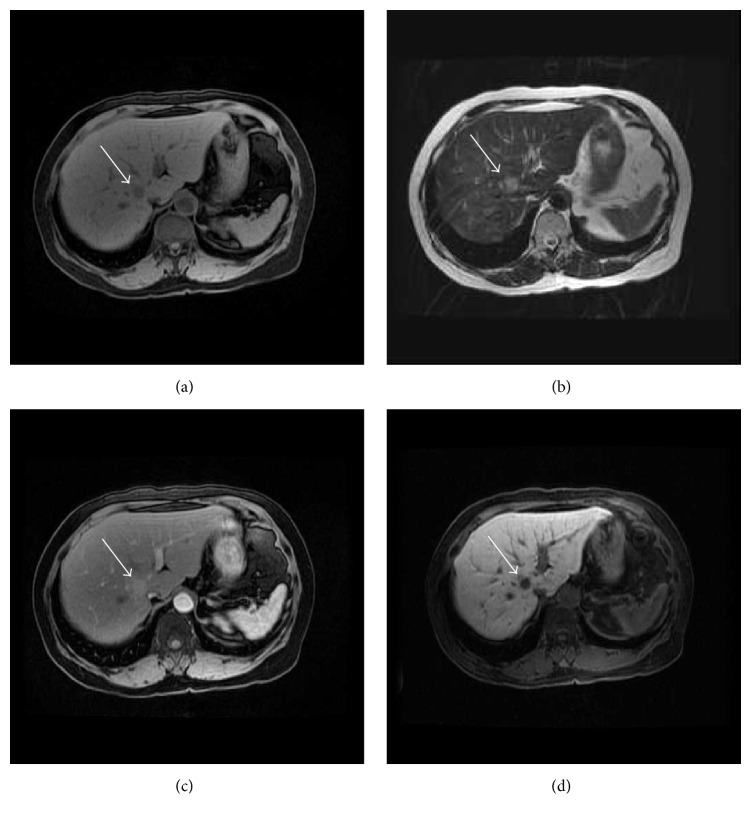
Magnetic resonance imaging. T1-weighted fat suppressed imaging showed low intensity at the tumor of S7 (arrow) (a). T2-weighted imaging showed high intensity at the tumor (b). In Gd-EOB-DTPA enhanced MRI, the tumor was enhanced in the arterial phase (c), and there was low signal intensity in the hepatobiliary phase (d).

The US revealed a 14 mm well-defined, hypoechoic mass in the posterosuperior subsegment of the right hepatic lobe (segment 7). Enhanced computed tomography (CT) revealed a 17 mm well-defined tumor that was enhanced in the arterial phase and washed out in the portal and delayed phase (Figure 1). On magnetic resonance imaging (MRI), T1-weighted fat suppressed imaging showed low intensity at the tumor of S7. T2-weighted imaging showed high intensity at the tumor. In Gd-EOB-DTPA enhanced MRI, the tumor was enhanced in the arterial phase, and there was low signal intensity in the hepatobiliary phase (Figure 2).

Based on the above findings, HCC was suspected. We performed a subsegmental resection of the liver.

**Figure 3:**
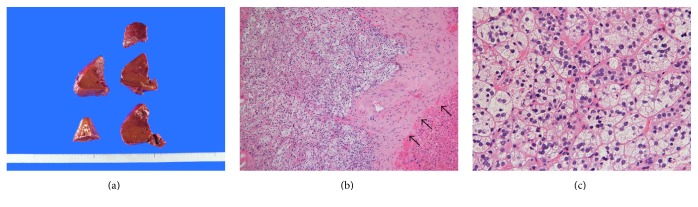
Macroscopic and pathological examinations. The lesion was not recognized by macroscopic examination of the cut surface of the surgically resected liver (a). Microscopically, the tumor is surrounded by the thin fibrous capsule (arrow) (b) (Hematoxylin and Eosin stain ×100). The lesion was composed of epithelioid cells with a microvesicular cytoplasm that formed nests or cords with abundant capillary vessels (c) (Hematoxylin and Eosin stain ×400).

The macroscopic findings revealed that the tumor was visually not apparent (Figure 3(a)). Microscopic findings showed that the tumor was surrounded by thin fibrous capsule (Figure 3(b)). The lesion was composed of cells with a microvesicular cytoplasm, and these were aligned in alveolar or trabecular arrangements in a similar manner to features of the adrenocortical tissue (Figure 3(c)). Therefore, we thought it was not HCC, suggesting the possibility of an adrenal rest tissue of the liver or a metastatic clear cell carcinoma of the kidney, although no mass was detected radiologically in the kidney, and a malignant tumor was not detected in the adrenal cortex.

**Figure 4:**
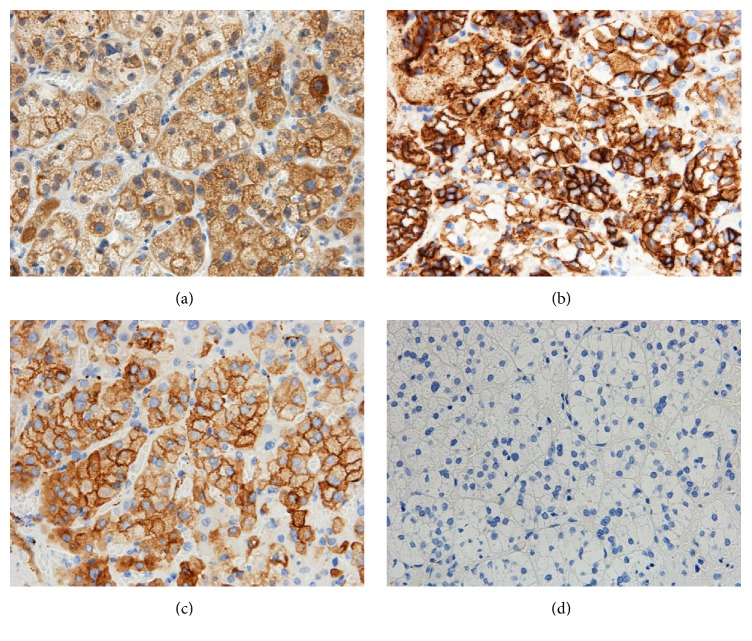
Immunohistochemical staining. Immunohistochemistry of the lesion. The cells are positive for inhibin *α* (a), CD56 (b), and synaptophysin (c), whereas cells were negative for chromogranin A (d) (all photomicrographs ×400).

Thus, we performed immunohistochemical staining. The tumor did not stain for liver marker Hep-per-1, AFP, and glypican-3, which are usual markers for HCC, and it did not stain for CD10, which is a diagnostic marker of clear cell renal cell carcinoma. Additionally, the cells were negative for cytokeratins (AE1/AE3, CAM5.2, CK7, and CK19). In contrast, positive staining was observed for inhibin *α*, melan A, synaptophysin, and CD56 but not chromogranin A (Figure 4). The Ki-67 proliferation index was less than 1% in the tumor cells.

Thus, the final histopathologic diagnosis in this case was an adrenal rest tumor of the liver.

## 3. Discussion

Most of the ectopic adrenal tissues are reported in relation to the kidney, and less than 100 cases have been reported near the genital structures [1, 2]. However, the incidence of ectopic adrenal tissue in the liver is exceedingly rare.

Embryologically, the adrenal cells have different embryological origin: the cortex is derived from the mesoderm and the medulla from the ectoderm of the neural crest. The fatal cortex is formed during the fourth and fifth weeks from the mesoderm, which lines the posterior abdominal wall. Cells from the neural crest invade the primitive cortex to form the medulla. Encapsulation of the medulla occurs late in fetal development [1, 12, 13]. It is postulated that adrenal rests within the genital structures were due to mechanical separation and displacement of portions of cortical tissue and associate with gonadal tissue during its descent. Bozic suggested that the unusual location of ectopic tissue may be related to misplaced mesothelial cells or autonomous differentiation of mesodermal elements [14].

The clinical significance of ectopic adrenal tissue is usually minimal and typically not associated with endocrine abnormalities. Only two cases were described as functional hepatic adrenal rest tumors, which can cause problems, including hyperplasia and neoplasia [3, 4, 15].

An accurate preoperative radiological diagnosis is very difficult. An adrenal rest tumor usually appears as a round, well-defined mass in the right hepatic lobe. One specific radiological diagnostic character is the presence of fat components [6]. This will reflect intracytoplasmic lipid droplets of the tumor cells. The other diagnostic character is hypervascularity. The hepatic adrenal rest tumor is mainly supplied by the hepatic artery [7]. At dynamic contrast-enhanced CT or MRI, angiomyolipoma also demonstrates early intense contrast enhancement [16]. MRI typically demonstrates the fat component and prominent central vessels. In this case, it is important to differentiate between adrenal rest tumor and early HCC. The typical MRI appearance of early HCC is iso- to hyperintense on T1-weighted images and hypointense on T2-weighted images [17]. Early HCC shows hypointensity in the hepatobiliary phase in Gd-EOB-DTPA enhanced MRI [18]. In our case, the remarkable radiological findings were hypervascularity in the arterial phase and washout in the delay phase, which was similar to HCC. However, T2-weighted imaging showed high intensity at the tumor. As MRI findings were not typical, we should try biopsy.

Macroscopically, a hepatic adrenal rest tumor is a well-circumscribed, yellowish, nodular lesion, which usually occurs under the capsule of the right hepatic lobe [19]. The pathologic appearance of this tissue is characteristic. The findings consist of cord-like arrangements of round to polygonal, pale, lipid-rich cells separated by vascular channels or bands of collagen [5]. However, Sugiyama reported that it is difficult to distinguish between hepatic adrenal rest tumor and HCC on the basis of histologic analysis alone [11]. Immunohistochemistry showed the tumor cells to be weakly stained for Hep-per-1, which can be considered a specific marker for HCC. Synaptophysin, inhibin, and CD56 are thought to be sensitive markers for adrenal cortical cells. In particular, CD56 is an excellent marker for distinguishing adrenal cortical cells from hepatocytes [20]. In this case, due to the influence of operative hemorrhage, we could not identify the tumor macroscopically. Nevertheless, microscopic findings showed the characteristic features of cortical adrenal tissue. Immunohistochemistry shows that the tumor in our case was negative for HCC markers and positive for adrenal cortical cell markers. Therefore, we considered this tumor to be an adrenal rest tumor.

## 4. Conclusions

We described a rare case of adrenal rest tumor of the liver. Adrenal rest tumors are rare and uncommonly found in the liver. This tumor was similar to HCC in the radiological findings. A reliable diagnosis is difficult by radiological findings alone. Adrenal rest tumors should be added to the list of radiological differential diagnoses of hypervascular hepatic tumors.
